# Commencement Week at the Baltimore College of Dental Surgery

**Published:** 1868-04

**Authors:** 


*Commencement Week at the Baltimore College of Dental Surgery.*

THE examination of the candidates for graduation commenced on Thursday, March 5th, and were concluded by the 10th, on the evening of which day the result was announced by the Dean, to the class assembled in the Chemical Hall.

On the morning of the 10th, Dr. L. D. Shepard, one of the "Harris Lecturers" delivered the first of his lectures on "Mallet Filling," the subject being "The Theory of Mallet Filling."

On the morning of the 11th Dr. Shepard delivered his second lecture, the subject of which was "The Practice of Mallet Filling," and during the afternoon, and also on the morning of the 12th, held clinics in the Infirmary of the College.

These lectures were very interesting, and were highly appreciated by the students, while the operations performed in the clinics, were of such a character as to confirm the high reputation of the operator. Earlier in the session Dr. James B. Bean, also one of the "Harris Lecturers" delivered several lectures on the "Aluminum Base," describing the process in such a lucid manner as to make his remarks very interesting and instructive. Dr. B. F. Arrington also delivered a lecture on the morning of the 7th of March on "Practical and Conservative Dentistry," which was listened to with great satisfaction and benefit by all present.

On the evening of the 12th of March the *Twenty-Eighth Annual Commencement* was held in the Concordia Opera House. Notwithstanding the unfavorable state of the weather the hall was densely filled by a fashionable and brilliant audience, a large majority of whom were ladies, nearly every one bore in their hands floral tokens of regard, such as bouquets, wreaths or baskets, which were bestowed upon their friends among the graduates. The Blues Band occupied the orchestra and during the evening discoursed many pleasing airs, as was testified by the applause of the audience.

After the faculty, graduates and guests had taken their seats upon the stage, the exercises were commenced with prayer by the Rev. Dr. Leyburn, which was followed by the reading of extracts from the charter of the College by the Dean, Professor F. J. S. Gorgas, who also after announcing the graduates, conferred upon the following gentlemen, twenty-seven in number, the degree of Doctor of Dental Surgery.

Newton M. Burkholder........................Virginia.
Edwin Z. Buchen..............................Maryland.
W. Leigh Burton..............................Virginia.
William Sale Carruthers....................Texas.
Benjamin F. Cosby...........................Virginia.
Robert Thomas Couch........................Virginia.
David William Crowther.....................Alabama.
Charles Gray Edwards.......................Virginia.
Langston James Goree.......................Texas.

Charles Wilson Harris.........................Virginia.
George Thomas Harris.........................Virginia.
John W. Holt.....................................North Carolina.
John Keys, M.D.................................Virginia.
Edwin Walter L'Engle.........................Florida.
George Allan McIntyre........................Florida.
Albert Ship McLeod...........................South Carolina.
Edward Coale McSherry......................Maryland
William Bailey Murphy.......................North Carolina.
Alexander Lee O'Brien.......................Virginia.
Rufus Hannibal Reeves.......................Tennessee.
John Doke Scott, M.D........................Virginia.
John Franklin Setzer..........................North Carolina.
George Alsop Sprinkel........................Virginia.
Thomas J. Thomas..............................Cuba.
Reuben Bossart Weiser........................Illinois.
Basil Manly Wilkerson........................Alabama.
Edgar McClintick Williams..................West Virginia.

Of which number there were from

Maryland, 2; Virginia, 11; West Virginia, 1; North Carolina, 3; South Carolina, 1; Tennessee, 1; Alabama, 2; Texas, 2; Illinois, 1; Florida, 2; Cuba, 1.

The Valedictory Address, which was very interesting and appropriate, and which appears in the present number of the Journal, was delivered by Henry R. Noel, M.D., Professor of Physiology. Following this address, was the presentation of a valuable gold-headed cane by Mr. Judson B. Wood of Virginia, on behalf the Junior class, to Dr. Hugh McGinnis Grant, Demonstrator of Operative Dentistry; the presentation was prefaced with some very appropriate remarks, which were responded to by Dr. Grant in a pleasing and able manner.

Professor Thomas E. Bond yielding to the repeated calls of the audience as well as the graduating class, made some pointed and amusing remarks, after which the benediction was pronounced by the Rev. Dr Leyburn. All the invited guests on the stage expressed themselves as highly pleased with the ceremonies of the evening; every thing passed off in a very pleasant manner, rendering this one of the most agreeable commencements ever held in Baltimore. The number of students in attendance on the lectures during the past session was sixty-nine.

The *twenty-ninth Annual Session* will commence on the 15th of October next.

